# The Synthesis of ^3^H-Labelled 8-Azido-N^6^-Benzyladenine and Related Compounds for Photoaffinity Labelling of Cytokinin-Binding Proteins

**DOI:** 10.3390/molecules24020349

**Published:** 2019-01-18

**Authors:** David. S. Letham, Xue-Dong Zhang, Charles H. Hocart

**Affiliations:** 1 Research School of Biology, Australian National University, Canberra ACT 0200, Australia; donxdzhang@yahoo.com.au (X.-D.Z.); Charles.Hocart@anu.edu.au (C.H.H.); 2 School of Chemistry and Chemical Engineering, Shaanxi University of Science and Technology, Long Shuo Rd, Wei Yang District, Shaanxi 710021, China

**Keywords:** 8-azido-N^6^-benzyladenine, cytokinins, cytokinin-binding proteins, receptors, photoaffinity labelling, synthesis of cytokinin analogues

## Abstract

The biology of the group of plant hormones termed cytokinins is reviewed to reveal areas where further studies of cytokinin-binding proteins could be significant. Such areas include: inhibition of human tumour cell growth by cytokinin ribosides, the role of cytokinins in the development of diverse micro-organisms including the cyanobacteria and *Mycobacterium tuberculosis*, the very rapid responses of plant cells to exogenous cytokinins, and other aspects of cytokinin plant biology. Photoaffinity labelling (PAL) coupled to the recent advances in HPLC of proteins and mass spectral analysis and sequencing of proteins, may have relevance to these areas. To facilitate PAL, we present experimental details for two methods for synthesis of 8-azido-N^6^-benzyladenine, which has the azido affinity group in the preferred position of the purine ring. Synthesis from [2-^3^H]adenosine yielded the above-mentioned PAL reagent with ^3^H in the purine ring and also gave labelled 9-riboside and 8-azido-N^6^,9-dibenzyladenine. 8-Azido-N^6^-benzyladenine was also prepared from 6,8-dichloropurine by a facile synthesis, which would allow a label to be sited in the benzyl group where substituents can also be introduced to vary cytokinin activity. The use of inactive cytokinin analogues in assessing the significance of PAL is discussed.

## 1. Introduction

### 1.1. Binding of Cytokinins as Plant Regulators

The cytokinins, N^6^-substituted adenines, are a group of plant hormones that regulate many features of plant development including cell division, leaf senescence, lateral shoot outgrowth and nutrient translocation [1,2]. The natural cytokinins are of two types [3]: those with an isoprenoid N^6^ substituent (e.g., zeatin, *E*-6-(4-hydroxy-3-methylbut-2-enylamino)-purine and N^6^-isopent-2-enyladenine termed iP), and those with a benzyl or substituted-benzyl N^6^-substituent (e.g., N^6^-benzyladenine, BA; 6-(*o*-hydroxybenzylaminopurine, termed *o*-topolin). The cytokinins occur as free bases, 9-ribosides, riboside-5′-phosphates, and as glucosides (sugar at position 3, 7 or 9 of purine ring and at *O* of zeatin). However, the free bases appear to be the active form in plants and bind to receptors [4].

Three histidine kinase cytokinin receptors (AHK2, AHK3, and AHK4 or CRE) have been characterized in *Arabidopsis* [5] and three similar histidine kinase receptors have been defined in maize [6]. Both *Arabidopsis* and maize receptors appear to be predominantly localized in the ER and the former are active in signal transduction [7]. Other similar receptors remain to be characterized in higher plants [8,9] in which several binding proteins of uncertain significance have also been detected. However, the rapid responses to exogenous cytokinin by isolated mitochondria suggest significant binding that does not involve known receptors that regulate transcription [10]. The effects on oxygen consumption, for example, are almost immediate. Rapid cytokinin effects on protein synthesis in mitochondria and plastid preparations have also been recorded, as well as very rapid opening of stomata [11].

In some situations, cytokinins may act at the site of biosynthesis, but these regulators can also move from a site of synthesis to one of action and thus conform to the traditional definition of a phytohormone. The view that root-produced cytokinin moves in the xylem to control numerous phases of shoot development has been confirmed by recent evidence including: (1) root nodules that overproduce cytokinins [12]; (2) supply of endogenous xylem cytokinins to excised monocot leaves at natural flux rate [13]; and (3) grafting of normal roots to mutant shoots deficient in cytokinins [14,15]. Zeatin-type cytokinins predominate in xylem, but the isopentenyl type are dominant in the phloem sap moving to control pattern development in the root [16]. This selective loading into xylem and phloem must involve binding of cytokinins to specific trans-membrane transporter proteins and details of this control are now emerging [17]. In contrast to this long-distance translocation, cytokinin translocation within seeds is important in germination. Isoprenoid cytokinins in dry lupin seed, for example, are completely degraded during imbibition [18], but cytokinins subsequently synthesized in the embryonic axis [19] move to the cotyledons [20] to induce expansion, enzyme activities and chloroplast formation [21,22]. The role of cytokinin binding proteins and receptors in seed germination is not known. Proteins analogous to the cereal embryo proteins that bind and possibly stabilize cytokinins, could be involved.

### 1.2. Cytokinins as Inhibitors of Tumour Cell Growth

Considerable interest now centres on the ability of cytokinin ribosides to inhibit the growth of human cancer cells in culture and cause apoptosis [9,23,24]. In a study of nearly all naturally occurring cytokinins (over 40 compounds), the ribosides of iP, 6-furfurylaminopurine, BA and *o*-topolin were the most effective inhibitors, showing cytotoxicity at 0.5–5 µM in 7 of 9 tumour cell lines [25]. The 2,6,9-trisubstituted purines are the other group of compounds related to cytokinins that inhibit tumour cell growth. The prototype was 2-(2-hydroxyethylamino)-6-benzylamino-9-methylpurine, which was originally designed and synthesized [26] as an inhibitor of a radish cotyledon enzyme that glucosylates cytokinin bases at positions 7 and 9 [27,28]. In a comparison of 81 purines as inhibitors of cyclin-dependent kinases (enzymes involved in regulation of the cell division cycle), this trisubstituted purine (now termed olomoucine) was found to be the most effective compound [29] and was also a potent inhibitor of tumour cell cultures. Determination of the mechanism of action (competitive inhibitor of ATP, non-competitive for histone H1) [29], resulted in the design of closely related compounds with enhanced inhibitory activity, one of which (roscovitine) is now in phase II CLINICAL trials as an anti-cancer drug.

The mechanism of action of cytokinin ribosides as tumour cell inhibitors is not clear. Elucidation may similarly lead to new cancer chemotherapies. Identification of cytokinin, and especially cytokinin riboside binding proteins in tumour cells, is relevant in this regard.

### 1.3. Cytokinins and Microorganisms

A growing number of microorganisms have been found to produce cytokinins that are recognized by the AHK-type receptors in intact plants. It is well established that plant microbial pathogens use this signal to the plant to modify plant organ development and to increase host susceptibility, thus facilitating infection [30]. However, recent studies indicate that this signal direction can be reversed. Thus, partially characterized receptors resembling the plant AHK proteins have been detected in phytopathogenic bacteria and certain fungi, and also in cyanobacteria [31]. These bacteria and fungi appear able to recognise cytokinin derived from plants.

In a recent advance, cytokinins were found to be produced by certain human microbial pathogens, which also responded to applied cytokinin. Exogenous zeatin promoted cell cycle progression in an obligate protozoan pathogen, which also produced zeatin and iP in nucleotide form [32]. However, a receptor was not identified and a plant-type AHK receptor gene was absent suggesting the presence of a new receptor type. A further observation of great interest is the secretion of several isoprenoid cytokinins by *Mycobacterium tuberculosis* [33] which occurs naturally only in humans. The biosynthesis and function of these cytokinins are unknown, but by analogy with plant systems, it has been suggested that the cytokinins signal transcriptional changes in human cells to favour growth of the bacteria. Exogenous iP induced transcriptional changes that altered the bacterial cell envelope showing that *M. tuberculosis* responds to change in cytokinin level [34]. *M. tuberculosis* also contains a homologue of the plant cytokinin activating enzyme LOG recently detected in other human pathogens including *Staphylococcus aureus* (also termed “golden staph”). Thus, cytokinins appear to be recognized as regulatory molecules in all the above microorganisms, opening possible strategies for pathogen control and a new field for studies of cytokinin-binding proteins. Cytokinins have roles beyond plant development that are yet to be characterized.

### 1.4. Cytokinins and Photoaffinity Labelling

Photoaffinity labelling (PAL, also denotes photoaffinity label) is a technique which could facilitate the identification of cytokinin receptors and binding proteins in the diverse biologies already identified. It has been used very successfully in drug research to identify receptors and depends on the presence of a photoactivated group attached to the ligand [35]. The method can be applied to cytokinin analogues with an azido group inserted on an aromatic ring. When such an analogue binds to a receptor or binding protein, irradiation with UV light converts the N_3_ group into a nitrene that inserts into any adjacent C–H, O–H or N–H bond, forming a covalent linkage with the binding protein [36]. The identification of PAL protein has been a major problem, which has now been greatly simplified by developments in the HPLC of proteins coupled with mass spectral methods for protein analysis and sequencing.

Certain synthetic phenylureas exhibit cytokinin activity. Tritium-labelled azido derivatives of these ureas [37,38], as well as labelled 2-azido-BA [39,40] have been used in PAL to identify some cytokinin-binding proteins in higher plants. However, these proteins are not functional receptors and their significance is obscure. For PAL, 2-azido-purine cytokinins have an unsatisfactory feature. These compounds are in equilibrium with isomers formed by ring closure of the azido substituent to N-1 and N-3 (the azidoazomethine-tetrazole equilibrium in 2-azido-purines [41]). This appears to be the cause of the undesirably long period required for photolysis, during which, secondary photolytic reactions and unspecific labelling would occur.

Properties of 8-azido-N^6^-substituted adenines differ from those of the corresponding 2-azido compounds in two important respects relevant to PAL. Firstly, the 8-azido compounds are markedly more effective as cytokinins. Thus, in the tobacco tissue bioassay while 2-azido-BA has activity similar to that of BA, 8-azido-BA is “over 25 times as active” as BA [42] (the least detectable concentrations are about 3 × 10^−5^ and 10^−3^ µM respectively). Secondly, the 8-azido compounds do not require extended irradiation for photolysis. These two properties of 8-azido cytokinins would be expected to minimize unspecific labelling.

Detection of PAL-proteins requires a label in the probe. Biotin is often used in this way and it also facilitates purification of the labelled protein. However, linkage to biotin would greatly reduce or eliminate the activity of a purine cytokinin. Labelling with radioactivity appears to be necessary, preferably tritium to give high specific activity.

Accordingly, in this paper, we discuss the biological areas to which PAL with cytokinins would be relevant. The synthesis of ^3^H-labelled 8-azido-BA and also that of the corresponding riboside are described, providing the first syntheses of these tritium-labelled compounds. The role of azido derivatives of inactive cytokinin analogues in the assessment of PAL binding is also discussed.

## 2. Results and Discussion

### 2.1. Possible Synthetic Routes to [^3^H]8-Azido-BA

Unlabelled 8-azido-BA has been synthesized by three methods:

1. From 8-bromo-adenosine by conversion to 8-azido-adenosine, followed by benzylation and ribose cleavage [43].
2. From 6,8-dichloropurine by four steps including blocking and deblocking N-9 [42].
3. By direct bromination of BA and reaction with sodium azido under novel conditions [44].

**Figure 1:**
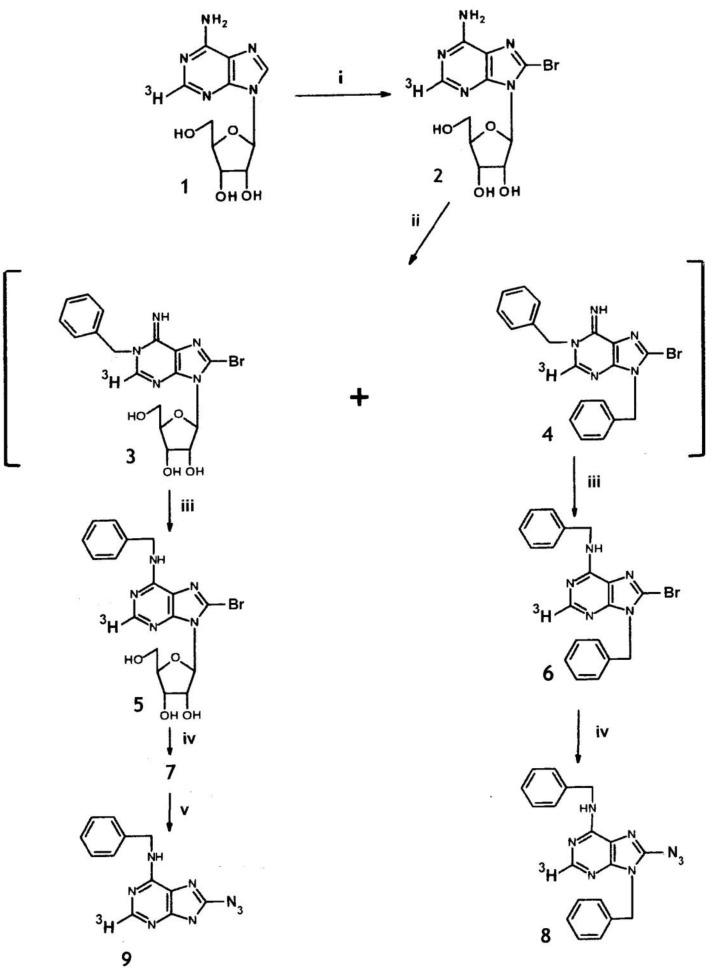
The sequence of reactions used to prepare photoaffinity compounds from [2-^3^H]adenosine. The compounds and intermediates are numbered **1** to **9** as in the text. The 1-substituted adenosine derivatives, **3** and **4,** were not preparatively separated but the mixture was subjected to conditions to induce a Dimroth rearrangement yielding the N^6^-derivatives **5** and **6**. The reactions were (i) bromination; (ii) reaction with benzylbromide in DMF; (iii) rearrangement in 0.25 M NH_4_OH; (iv) NaN_3_ at 70 ^o^C; and (v) 0.5 M HCl at 80 ^o^C.

These methods involve macro-synthesis using gram amounts of compounds and purification by classical methods. When using ^3^H-labelled starting compounds, micro synthesis from a few mg or less is necessitated by the cost of ^3^H-labelled precursor. The purification methods given are therefore not directly applicable to the synthesis of [^3^H]8-azido-BA. Furthermore, the yield of unlabeled compound by method (1) was only 6% from 8-bromo-adenosine [43]. [^3^H]Adenosine is available commercially at high specific activity to prepare 8-bromo-adenosine, but benzylamine and BA at this activity for methods (2) and (3) respectively are not available. Provided adenosine can be brominated to yield the 8-bromo-derivative in high yield by micro-synthesis, and the low reported yield in its conversion to 8-azido-BA could be elevated, synthesis of this photoaffinity label from [^3^H]adenosine becomes the preferable route, since it also yields the riboside needed for studies of tumour cells. This was achieved in the optimized micro-synthesis discussed below and outlined in Figure 1. For the synthesis, an excess of unlabeled adenosine was added to the high specific activity [^3^H]adenosine.

### 2.2. Synthesis of 8-Azido-N^6^-benzyl-[2-^3^H]adenine from [2-^3^H]Adenosine

**Figure 2:**
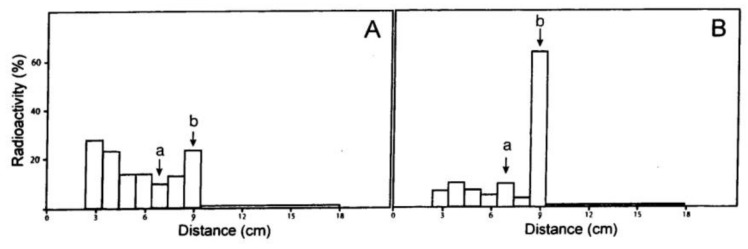
The distribution of radioactivity over the TLC chromatograms show the effects of temperature and solvent on bromination of [2-^3^H]adenosine. (**A**) water at 26 ^o^C (conditions often used to brominate nucleotides); (**B**) 50% methanol at 4 ^o^C (conditions used herein). The quantities used in the reactions are those given in Section 3.3.1. The letters a and b denote adenosine and 8-bromoadenosine, respectively. For both graphs, the reaction time was 0.75 h and solvent A was used for TLC.

The first step was bromination of adenosine (**1**) at C-8. Selective bromination at the purinyl C-8 of some nucleotides has been successful using water as solvent at 23 °C. However, TLC studies indicated that the yield of [2-^3^H}8-bromo-adenosine (**2**) by bromination at 4 °C in 50% methanol with suspended CaCO_3_ markedly exceeded that obtained under the above conditions and in other solvents (cf. Figure 2).

When **2** was benzylated with benzyl bromide in dimethylformamide (DMF) at 37 °C, the reaction was slow and a significant amount of product was identified as a dibenzyl derivative. At higher temperature (60–85 °C), the ratio of di- to mono-benzyl products was enhanced, but much reduced at 23 °C with a slow reaction rate. However, the reaction can be catalyzed by the addition of CaCO_3_. Under these conditions, **2** was almost completely benzylated in DMF by benzylbromide in 3 days, resulting in a mixture of 1-benzyl-8-bromo-adenosine (**3**, the expected product [45]) and 1, 9-dibenzyl-8-bromo-adenine (**4**) in a ratio of 3:1. The mixture was dissolved in 0.5 M NH_4_OH at 37 °C and kept for 36 h to induce a Dimroth rearrangement (conversion of 1-substituted adenine to the N^6^ isomer [46]) yielding N^6^-benzyl-8-bromo-[2-^3^H]adenosine (**5**) and N^6^,9-dibenzyl-8-bromo-[2-^3^H]adenine (**6**).

For direct displacement of leaving groups (e.g., Br) from the C-8 position of purines, the acidic 7(9) proton usually requires masking [42]. In the present situation, this was achieved by the ribosyl moiety in the case of **5** and benzyl in **6**. Hence, these 8-bromo compounds were reacted with an excess of NaN_3_ in DMF at 70 °C yielding the corresponding azides **7** and **8**, respectively, which were separated from any unreacted bromide by HPLC (separation not achievable by TLC). Cleavage of the ribose moiety from **7** by acid hydrolysis yielded [^3^H]8-azido-BA, i.e., 8-azido-N^6^-benzyl-[2-^3^H]adenine (**9**). The overall yield from [2-^3^H] adenosine was 22%; the specific radioactivity of **9** was 21.8 MBq/µmol; and the radiochemical purity (>98%) was established by normal TLC, reversed phase TLC and HPLC. The MS of **9** confirmed the assigned structure with prominent ions at *m*/*z:* 226 (M^+^), 238 (–N_2_ from azido), 237 (further loss of H), 161 (loss of C_6_H_5_-CH=NH from M^+^ with H transfer to purine fragment ion). Because of the molar excess (30-fold) of the unlabeled adenosine added to the [^3^H]adenosine for the synthesis, the plotted MS for the above compounds fail to reveal the [^3^H]-labelled ions.

### 2.3. Synthesis of 8-Azido-N^6^-Benzyladenine from 6,8-Dichloropurine

**Figure 3:**
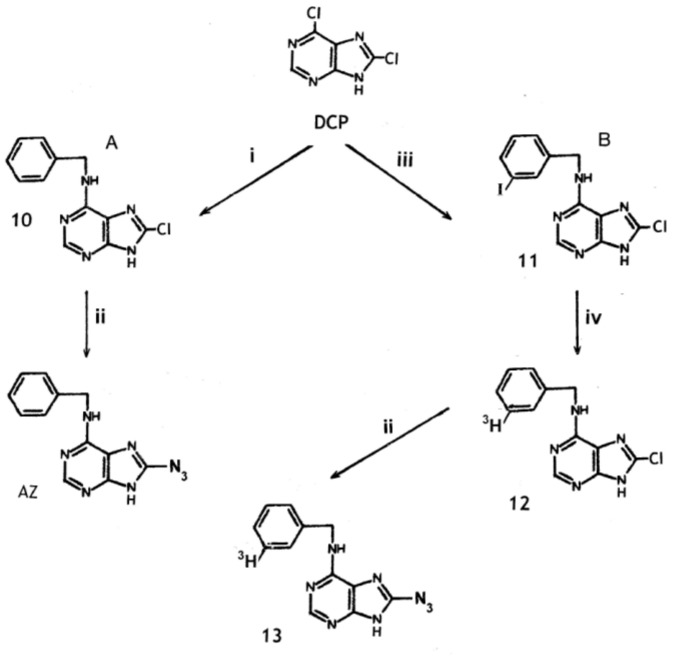
Reaction sequences for synthesis of 8-azido-N^6^-benzyladenine from 2,6-dichloropurine (DCP). A. Synthesis of the unlabelled compound (AZ) as described herein. B. Proposed synthesis of ^3^H-labelled compound of high specific activity, i.e., 8-azido-N^6^-[3-^3^H]benzyladenine (**13**). Intermediates shown in the figure are: N^6^-benzyl-8-chloro-adenine (**10**), 8-chloro-N^6^-(3-iodobenzyl)adenine (**11**), N^6^-[3-^3^H]benzyl-8-chloro-adenine (**12**), 8-azido-N^6^-[3-^3^H]benzyladenine (**13**). The reactions were with: (i) benzylamine at 100 ^o^C; (ii) NaN_3_ in propan-1-ol containing acetic acid; (iii) 3-iodobenzylamine; (iv) tritium gas over palladium catalyst.

Synthesis of the unlabeled compound is considered first. In an analogous situation, 2,6-dichloropurine was found to react with NaN_3_ to yield the 2,6-diazo compound, which was then reacted with benzylamine to yield 2-azido-BA [42]. However, we found that this reaction sequence did not convert 6,8-dichloropurine into 8-azido-BA. The first step in this synthesis from 6,8-dichloropurine requires formation of N^6^-benzyl-8-chloro-adenine (**10**). In previous studies, replacement of the chlorine in **10** by the azido group required prior blocking of the N-9 position followed by reaction with triethylammonium azide. Deblocking then yielded 8-azido-BA [42]. A simple direct method for this conversion is desirable, especially when ^3^H-labelled compounds are involved, and is detailed in Section 3.4. It involves the reaction of **10** directly with excess NaN_3_ in propan-1-ol containing acetic acid at 100 °C. The acid promoted the reaction probably by suppression of the ionisation of the 7/9 proton and formation of the 9-N anion [42]. This reaction provided a direct synthesis of unlabelled 8-azido-BA (Figure 3). However, it also allows the introduction of a label into the benzyl group if labelled benzylamine was available for the synthesis of the precursor **10**. The amine is available commercially labelled with ^14^C, while catalytic reduction of iodobenzylamine with ^3^H gas should give high-specific-activity labelling in the benzene ring. Reduction of benzonitrile with AlCl_3_-activated [^3^H]NaBH_4_ [47] gives methylene labelling of low specific activity (unpublished data). However, the optimum ^3^H-labelling of 8-azido-BA, based on synthesis from 6,8-dichloropurine, probably involves formation of 8-chloro-N^6^-(3-iodobenzyl)adenine (**11**) for catalytic reduction with tritium gas at room temperature giving deiodination and yielding N^6^-[3-^3^H]benzyl-8-chloro-adenine (**12**) (Figure 3). Based on the results of an analogous reaction [48]; note, this paper describes the synthesis of N^6^-[3-^3^H]benzyl-2-chloro-adenine. In unlabeled form, this compound was previously converted to 2-azido-BA through formation of the 2-hydrazino derivative], a specific radioactivity of about 5-6 Ci/mmol would be expected for **12** and the resulting 8-azido-BA (**13**).

As an alternative to the above methods, catalytic exchange with ^3^H gas at elevated temperatures has been applied to label cytokinins [49] and may be applicable to **10,** which may exhibit sufficient stability because it lacks an azido group.

Some compounds that are potentially useful in the synthesis of di-azido derivatives of BA were also prepared (see Experimental), namely: N^6^-benzyl-2,8-dichloro-9-(tetrahydropyran-2-yl)adenine (A), 2,6,8-triazido-purine (B), and 2,6,8-trichloro-9-tetrahydropyran-2-yl)purine which was converted to the 9-(tetrahydropyan-2-yl) derivative of B (termed C) by reaction with NaN_3_. A (by reaction with NaN_3_), while B and possibly C (by reaction with benzylamine) could provide direct routes to 2,8-diazdio-N^6^-benzyladenine. The presence of two azido groups may facilitate the PAL reaction.

### 2.4. Assessment of PAL

It is often suggested that when an active cytokinin competes with a cytokinin carrying a PAL at a binding site, the site is involved in cytokinin action and could contain a functional receptor. In this connection, it would be relevant to study the PAL in the presence of an inactive cytokinin analogue that does not suppress the bioassay activity of active cytokinins. This analogue presumably does not bind to the relevant receptor. If PAL is suppressed, the binding site cannot be a functional receptor, but may be, for example, a metabolizing enzyme, or may have no relevance at all.

N^6^-(3,4-Dimethoxybenzyl)adenine (DMA) is a cytokinin analogue of the above type that was noted in our previous studies [50]. While BA showed activity at 10 nM in the radish cotyledon bioassay, DMA was inactive at 100 µM and did not suppress the activity of BA. DMA was also inactive in the *Amaranthus* betacyanin bioassay and very weakly effective in a wheat leaf senescence assay [51]. This BA derivative appears to have relevance to PAL in relation to radish cotyledon expansion and probably to other cytokinin-induced responses. It is also relevant that DMA did not activate the AHK3 and AHK4 receptors [51].

Very detailed compilations [9,51] of the activities of BA derivatives, including many newly synthesized compounds, show that several are nearly inactive in some cytokinin bioassays and also in activation of the AHK3 receptor. Thus, these compounds may resemble DMA in activity. Addition of such BA analogues prior to PAL with **9**, could yield significant conclusions, but synthesis of 2- or 8-azido derivatives of such inactive compounds for comparative PAL with 2- or 8-azido BA may be more significant as all compounds then have an azido group, which can directly promote cytokinin activity/binding [42]. A correlation between PAL labelling and cytokinin activity would be consistent with the binding site having a functional significance in terms of growth or physiological activity.

The 2-azido cytokinins mentioned above can be synthesized readily from 2,6-diazidopurine (the synthesis of 2-N_3_-DMA is detailed in the Experimental Section, while 2-N_3_-BA is a known compound [52]). By heating with tritiated water (readily available at 5 Ci/mL), these 2-azido compounds can be labelled by exchange of the C-8 proton [53] to give a low but still useful specific activity (maximum from above ^3^H-water, 45 mCi/mmol).

8-Azido-N^6^,9-dibenzyladenine (**8**), the tritium-labelled compound formed as a secondary product in the synthesis of **9** from adenosine, provides a further but structurally different compound to assess PAL with **9**. The benzyl substituent, like many other groups at N-9, markedly lowers cytokinin activity and would be expected to greatly reduce PAL at a receptor site.

## 3. Experimental Section

### 3.1. Chromatographic Methods

Unless stated otherwise, Merck silica gel PF_254_ was used for normal phase TLC and layers were developed in tanks lined with filter paper saturated with solvent. The solvents used were (proportions are by volume): (A) butan-1-ol/H_2_O/14 M NH_4_OH (6:2:1) upper phase; (B) chloroform/methanol (9:1); (C) chloroform/methanol (9:1) with 14 M NH_4_OH added to solvent in tank (0.2 mL/100 mL) and to paper linking tank (several drops); (D) chloroform/methanol/acetic acid (95:5:0.5); and (E) toluene/ethyl acetate/acetic acid (500:180:36). For reverse phase (RP) TLC, silica layers were impregnated with DMPS-5X fluid [54] and the solvent was methanol/H_2_O (20:80). Compounds separated on TLC plates were detected by short wavelength UV light. Azido compounds were streaked across the origin of TLC plates for preparative separation. After TLC, only the edges of the plates were exposed to UV light and these areas were discarded.

HPLC utilized a Waters system with effluent absorbance monitored at 265 and 280 nm. The column was an RCM 100 Nova-Pak C-18 cartridge; solvent 35% ethanol with 1% acetic acid, flow rate 2.4 mL/min.

### 3.2. UV and Mass Spectra

UV spectra in neutral solution were recorded in ethanol or aqueous ethanol. Spectra in acidic or basic solution were recorded in aqueous ethanol containing 0.05 M HCl or 0.1 M NH_4_OH, respectively. All UV spectra were determined with a Shimadzu-240 spectrophotometer (Shimadzu, Kyoto, Japan).

Mass spectra (MS) were obtained with a Finnigan 4500 instrument (Finnigan, San Jose, CA, USA) with samples introduced via the direct inlet probe. Electron impact (EI): 70 eV; source 150 °C. Chemical ionization (CI): methane reagent gas (1 torr); ionization energy 140 eV; source 100 °C.

### 3.3. Synthesis of 8-Azido-N^6^-benzyl-[2-^3^H]adenine from [2-^3^H]adenosine

#### 3.3.1. 8-Bromo-[2-^3^H]adenosine (2)

A solution in 50% methanol (1 mL) was prepared containing unlabelled adenosine (3.0 mg, 11.2 µmol) and [2-^3^H]adenosine (296 MBq, 766 GBq/mmol). Bromine (38.2 µmol, 195 µL of a 1% *v/v* aqueous solution) and CaCO_3_ (2 mg) were added to this solution and the mixture was stirred at 4 °C for 2 h, the optimal time determined in preliminary studies involving TLC (solvent A). The above reaction mixture was evaporated in vacuo and the residue was dissolved in 50% ethanol for preparative TLC on silica gel (solvent A), while the product eluting solvent was 50% ethanol. Small amounts of unreacted [^3^H]adenosine were recycled through bromination and TLC giving a combined yield for **2** of 86% (10.0 µmoles, 23.9 MBq/µmol). EI-MS *m*/*z* (rel. int.): 345/347 (M^+^, 1%), 315/317 (3), 256/258 (20), 242/244 (13), 213/215 (100) (loss of ribose), 186/188 (18), 135 (26). This spectrum and TLC comparisons with authentic compound established the identity of the product as 8-bromo-adenosine (**2**).

#### 3.3.2. N^6^-Benzyl-8-bromo-[2-^3^H]adenosine (5) and N,9-dibenzyl-8-bromo-[2-^3^H]adenine (6).

Benzylbromide (16 µL, 130 µmol) was added to the above dried 8-bromo-adenosine in dry DMF (0.5 mL) and the mixture was stirred with CaCO_3_ (3.0 mg) at 23 °C for 3 days. After addition of 10 µL of triethylamine, the reaction was evaporated in vacuo. TLC combined with UV spectra indicated a predominance of 1-substituted adenines (**3**, **4**) and to induce rearrangement to the N^6^-benzyl derivatives, the residue was dissolved in ethanol (1 mL) to which NH_4_OH (0.5 M, 1 mL) was added. After 20 h at 37 °C, rearrangement was complete as evidenced by TLC (solvent A) and the centrifuged product was subjected to preparative TLC in this solvent, yielding N^6^-benzyl-8-bromo-[2-^3^H]adenosine (**5**, 4.8 µmol) at R_f_ 0.46: UV (100% ethanol) λ_max_ 273 nm, λ_min_ 238 nm; EI-MS *m*/*z:* 435/437 (M^+^, 1.2%), 405/407 (1.4), 346/348 (11), 332/334 (8), 303/305 (74), 224 (94), 198/200 (10), 119 (9), 106 (100); CI-MS (CH_4_) *m*/*z:* 464/466 ([M + C_2_H_5_]^+^, 3%), 436/438 (MH^+^, 32), 304/306 (80), 226 (100). Yield of **5**: 48% for this step.

The above preparative TLC also gave a lesser component at R_f_ 0.83 identified as N^6^,9-dibenzyl-8-bromo-[2-^3^H] adenine (**6**, 1.4 µmol): UV(100% ethanol) λ_max_ 274 nm, λ_min_ 243 nm; EI-MS *m*/*z:* 393/395 (M^+^, 100%), 392/394 ([M − H]^+^, 66), 314 (33), 302/304 (90), 197/199 (10), 106 (96). Yield of **6**: 14% for this step.

#### 3.3.3. 8-Azido-N^6^-benzyl-[2-^3^H]adenosine (7) and 8-azido-N^6^, 9-dibenzyl-[2-^3^H]adenine (8)

Compound **5** above, was dissolved in DMF (2 mL) and all further procedures were conducted under red light or in darkness. After the addition of NaN_3_ (200 µmol), the solution of **5** was heated at 70 °C for 80 h to achieve a complete conversion to the azido derivative. Since TLC did not achieve resolution of the bromo and azido compounds, this conversion was monitored by HPLC (see 3.1). The retention times were: **5** (5.88 min), azido **7** (6.50 min). When the reaction was complete, the residue obtained by vacuum evaporation was dissolved in water (4 mL) and extracted 5 times with equal volumes of ethyl acetate. Evaporation of the extracts yielded 8-azido-N^6^-benzyl-[2-^3^H] adenosine (**7**). UV (50% ethanol): λ_max_ 289 nm, λ_min_ 255 nm. EI-MS *m*/*z*: 398 (M^+^, 22%), 368 (14), 309 (28), 295 (16), 266 (91), 238 (67), 237 (100), 224 (16), 161 (58). CI-MS (CH_4_) *m*/*z*: 439 ([M + C_3_H_5_]^+^, 3%), 427 ([M + C_2_H_5_] ^+^, 5), 399 (MH^+^, 49), 267 (84), 239 (71). Yield of **7**: 81% for this step.

Similarly, **6** was reacted with NaN_3_ yielding 8-azido-N^6^, 9-dibenzyl-[2-^3^H]adenine (**8**).

#### 3.3.4. 8-Azido-N^6^-benzyl-[2-^3^H] adenine (9)

The foregoing riboside (**7**) was hydrolyzed in 0.5 M HCl (1.5 mL) at 80 °C for 2 h. Hydrolysis was monitored by TLC (solvent B) and the hydrolysate was then adjusted to pH 8-9 by addition of solid KHCO_3_. Four extractions with ethylacetate (1.5 mL) yielded a product for preparative TLC (solvent B) on HF_254_ silica gel. The dominant zone (R_f_ 0.76) was eluted with 50% ethanol to give 8-azido-N^6^-benzyl-[2-^3^H]adenine (**9**) 2.57 µmol). UV in 50% ethanol, the neutral, acidic and basic spectra: λ_max_ 290, 298 and 294 nm respectively; λ_min_ 254, 251 and 258 nm, respectively. EI-MS *m*/*z*: 266 (M^+^, 100%), 238 (M^+^ − N_2_, 58), 237 (89), 161 (45), 147 (21), 135 (34), 119 (14). Yield of **9**: 66% for this step; overall 22%.

### 3.4. N^6^-Benzyl-8-Chloro-Adenine (10), Ddirect Azido Insertion

6,8-Dichloropurine (3 mg, 15.8 µmol) and benzylamine (8.5 mg, 79 µmol) in butan-1-ol (0.15 mL) were heated in a sealed vial at 100 °C for 6 h. After TLC (solvent C), elution of the main UV zone yielded N^6^-benzyl-8-chloro-adenine (**10**), 2.7 mg (66% yield). UV acid: λ_max_ 279 nm; UV base: λ_max_ 276 nm. EI-MS *m*/*z*: 259/261 (M^+^, 30%), 224 (4), 223 (4), 154 (-C_6_H_5_-CH=NH, 6), 106 (66), 91 (100).

In synthesis designed to use all the supplied benzylamine (e.g., when the amine is ^3^H labelled), a large excess of 6,8-dichloropurine is required as exemplified below. Benzylamine hydrochloride (1.6 mg, 11.2 µmol), 6,8-dichloropurine (6.9 mg, 36.3 µmol), purified butan-1-ol (0.14 mL) and triethylamine (15 µL) were heated in a sealed vial at 110 °C for 10 h. TLC analysis of the reaction product, involving ninhydrin application and UV characterization, indicated the benzylamine was completely reacted and that **10** was the main reaction product. The excess of unreacted 6,8-dichloropurine remained at the origin during preparative TLC (solvent C), and was thus readily separated from **10** which exhibited a low R_f_.

N^6^-Benzyl-8-chloro-adenine (**10**) (1.0 mg, 3.9 µmol), NaN_3_ aqueous solution (60 µL containing 140 µmol), propan-1-ol (540 µL), and acetic acid (30 µL) were heated at 100 °C in a sealed vial for 24 h. After addition of water (1.5 mL), the reaction mixture was extracted with three 1.5-mL volumes of ethyl acetate. Preparative TLC (solvent C, eluent ethanol) of the evaporated extracts yielded a product (0.81 mg, yield 79%; separable from **10** by TLC solvent E) identical to 8-azido-BA prepared from adenosine. Both compounds turned yellow on TLC plates under UV light, co-chromatographed and exhibited identical UV and mass spectra. UV in 95% ethanol, acidic spectrum: λ_max_ 298 nm, λ_min_ 252 nm, shoulder 225–234 nm; basic spectrum: λ_max_ 294 nm, λ_min_ 257 nm, shoulder 227–237 nm. EI-MS *m*/*z*: 266 (M^+^), 238, 237, 161, 147, 135, 119 as for **9**.

### 3.5. Additional Compounds

#### 3.5.1. 2,6,8-Triazidopurine

Triethylammonium azide (TAA) was prepared by ion exchange from sodium azide and the triethylammonium form of Dowex 50. 2,6,8-Trichloropurine (TCP, 7 mg) dissolved in dry DMF (0.25 mL) was heated at 80 °C with dried TAA (100 mg, 7-fold excess) for 5 h. The reaction mixture was partitioned between ethyl acetate and water and the aqueous phase was extracted with ethyl acetate. TLC of the combined ethyl acetate solutions (solvent D) revealed only one UV absorbing component (R_f_ rel. TCP = 1.93) and this gave a brown colour under UV light whereas TCP did not. The new compound was further purified by retention from 25% (*v*/*v*) methanol on a C_18_ column (40 µm, 0.5 g) and elution as a sharp fraction by 80% (*v*/*v*) ethanol containing acetic acid (1%) to yield 2,6,8-triazidopurine. EI-MS *m*/*z*: 243 (M^+^, 100%), 215 (48), 201 (29), 173 (17), 145 (68).

#### 3.5.2. 2,6,8-Triazido-9-(tetrahydropyran-2-yl)purine

2,6,8-Trichloropurine (30 mg) was dissolved in dioxane (0.4 mL). After the addition of 3,4-dihydropyran (80 µL) and formic acid (40 µL), the solution was heated at 85 °C for 60 min before all solvent was evaporated and the residue dissolved in chloroform. This was extracted with 0.5 M aqueous ammonia (3 double volumes, discarded) to remove traces of the chloropurine. Evaporation of the dried chloroform phase yielded 2,6,8-trichloro-9-(tetrahydropyran-2-yl)purine (yield 36 mg). EI-MS *m*/*z*: 306/308 (M^+^, 6%), 222/224 (15), 187/189 (two Cl present, 9%), 85 (100), 84 (28), 67 (16).

The foregoing tetrahydropyranyl derivative (16 mg) was dissolved in ethanol (0.80 mL) and heated at 75 °C in a stirred sealed vial with NaN_3_ (20 mg) for 80 min. The evaporated reaction solution was partitioned between chloroform and water, and the former phase was re-extracted twice with water. Evaporation of the dried chloroform solution yielded 2,6,8-triazido-9-(tetrahydropyran-2-yl)purine. EI-MS *m*/*z:* 327 (M^+^, 4%), 243 (40), 215 (25), 201(5) 85 (100), 84 (37), 67 (27).

#### 3.5.3. 6-Benzylamino-2,8-Dichloro-9-(tetrahydropyran-2-yl)purine

A propan-1-ol solution (10 mL) of trichloropurine (240 mg) and benzylamine (400 mg) was refluxed for 3 h. The evaporated solution was partitioned between ethyl acetate and water neutralized with HCl. The ethyl acetate phase was washed with water and subjected to preparative TLC (solvent D) yielding 6-benzylamino-2,8-dichloropurine. EI-MS *m*/*z*: 295 (M + 2, 64%), 293 (M^+^, 100%), 292 (38), 258 (19), 216 (8) 187 (12), 153 (11), 106 (84). The foregoing product was dissolved in dioxane and converted to the 9-(tetrahydropyran-2-yl) derivative by the procedure described in Section 3.5.2.

#### 3.5.4. 2-Azido-N^6^-(3,4-dimethoxybenzyl)adenine

For this synthesis, 2,6-diazidopurine [42] (85 mg, 421 µmol) was added to 3,4-dimethoxybenzylamine hydrochloride (1.2 mmol) dispersed in water (3 mL). After addition of triethylamine (0.25 mL), the mixture was heated at 100 °C for 5 h and then neutralized (pH 7) with 0.5 M HCl. The product precipitated at 4 °C over 3 days and was filtered off and washed with water (yield 70 mg). UV in 95% ethanol, the neutral, acidic and basic spectra were: λ_max_ 276 and 236, 280 and 236, 277 and 238 nm, respectively (two maxima for each spectrum); λ_min_ 254, 257 and 255 nm, respectively. EI-MS *m*/*z*: 326 (M^+^, 52%), 297 (27), 283 (56), 267 (28), 166 (29), 151 (100, dimethoxy-benzyl or tropylium ion), 135 (46), 119 (27).
